# Hematopoietic Stem Cell Transplantation—50 Years of Evolution and Future Perspectives

**DOI:** 10.5041/RMMJ.10162

**Published:** 2014-10-29

**Authors:** Israel Henig, Tsila Zuckerman

**Affiliations:** 1 Department of Hematology and Bone Marrow Transplantation, Rambam Health Care Campus, Haifa, Israel; 2 Bruce Rappaport Faculty of Medicine, Technion, Israel Institute of Technology, Haifa, Israel

**Keywords:** Adoptive immunotherapy, alternative donor, conditioning, graft-versus-host disease, graft-versus-leukemia, hematopoietic stem cell transplantation

## Abstract

Hematopoietic stem cell transplantation is a highly specialized and unique medical procedure. Autologous transplantation allows the administration of high-dose chemotherapy without prolonged bone marrow aplasia. In allogeneic transplantation, donor-derived stem cells provide alloimmunity that enables a graft-versus-tumor effect to eradicate residual disease and prevent relapse. The first allogeneic transplantation was performed by E. Donnall Thomas in 1957. Since then the field has evolved and expanded worldwide. New indications beside acute leukemia and aplastic anemia have been constantly explored and now include congenital disorders of the hematopoietic system, metabolic disorders, and autoimmune disease. The use of matched unrelated donors, umbilical cord blood units, and partially matched related donors has dramatically extended the availability of allogeneic transplantation. Transplant-related mortality has decreased due to improved supportive care, including better strategies to prevent severe infections and with the incorporation of reduced-intensity conditioning protocols that lowered the toxicity and allowed for transplantation in older patients. However, disease relapse and graft-versus-host disease remain the two major causes of mortality with unsatisfactory progress. Intense research aiming to improve adoptive immunotherapy and increase graft-versus-leukemia response while decreasing graft-versus-host response might bring the next breakthrough in allogeneic transplantation. Strategies of graft manipulation, tumor-associated antigen vaccinations, monoclonal antibodies, and adoptive cellular immunotherapy have already proved clinically efficient. In the following years, allogeneic transplantation is likely to become more complex, more individualized, and more efficient.

## INTRODUCTION

Hematopoietic stem cell transplantation (HSCT) is one of the most unique procedures in medicine. Today HSCT has become a standard of care for hematologic malignancies, congenital or acquired disorders of the hematopoietic system, and it is also applied as a therapeutic option in some of the solid tumors.^1^ During the last two decades HSCT use has expanded worldwide and evolved in its technology. Nowadays HSCT is employed for novel indications such as autoimmune and inherited metabolic disorders.^2^,^3^

Largely, HSCT can be divided into two types: 1) autologous where a patient donates the marrow stem cells to himself, and 2) a more elaborate and complex process of allogeneic HSCT where a patient receives the stem cell graft from a healthy donor. According to the European Registry of Hematopoietic Stem Cell Transplantations, in 2012 as many as 42% of all HSCT were allogeneic.^4^

In allogeneic HSCT a stem cell donor can be a matched related sibling or a haploidentical (partially matched) family relative. Stem cell grafts can also be obtained from an unrelated volunteer or from a cryopreserved cord blood unit. The number of volunteer donors and cord blood units available is constantly rising, and from 200,000 donors registered in 1989 it has now increased to more than 23 million.^5^

Allogeneic stem cell transplantation is performed by qualified medical staff in a facility with adequate conditions (patient’s environmental isolation, cellular processing transplantation laboratory, apheresis unit). Both medical and psychological patient preparation should be thorough yet prompt. Patient follow-up after the transplantation requires qualified management by a multidisciplinary team. As in solid organ transplantations, the process involves the preparation and care of a healthy donor volunteer who should undergo a medical procedure of stem cell collection. However, allogeneic HSCT is still associated with marked morbidity and mortality,^6^ and involves high costs.^7^,^8^

In the first several decades transplantations were developed in a few major institutes (Seattle and then Johns Hopkins). However, HSCT nowadays has become unique in medicine, due to the increased number of transplantations from unrelated donors, which could not have succeeded without international collaboration and the good will of people who are prepared to be volunteer donors for patients all over the world.

This review will focus mostly on allogeneic HSCT, its history, evolution, and future perspectives.

## HISTORY

The first human bone marrow transfusion was given to a patient with aplastic anemia in 1939.^9^ This patient received daily blood transfusions, and an attempt to raise her leukocyte and platelet counts was made using intravenous injection of bone marrow. After World War II and the use of the atomic bomb, researchers tried to find ways to restore the bone marrow function in aplasia caused by radiation exposure. In the 1950s, it was proven in a mouse model that marrow aplasia secondary to radiation can be overcome by syngeneic marrow graft.^10^ In 1956, Barnes and colleagues published their experiment on two groups of mice with acute leukemia: both groups were irradiated as anti-leukemic therapy and both were salvaged from marrow aplasia by bone marrow transplantation. The first group received a syngeneic marrow (from mice of the same strain); however, most of the mice died from leukemia relapse. The second group received an allogeneic marrow from a different strain; none of the mice in this group experienced disease relapse, but all the animals died from a “wasting syndrome.”^11^ In these experiments, three major principles of allogeneic HSCT were demonstrated: 1) the role of the preparative anti-leukemic regimen in HSCT, 2) the ability of the new engrafted immune system to prevent leukemia relapse, and 3) activity of the engrafted immune system against the recipient.

The first allogeneic HSCT (leading to its current status) was pioneered by E. Donnall Thomas and reported in the *New England Journal of Medicine* on September 12, 1957.^12^ In this study six patients were treated with radiation and chemotherapy and then received intravenous infusion of marrow from a normal donor. Only two patients engrafted, and all died by 100 days post the transplantation. At that time, little was known about histocompatibility antigens, and no one tried to match donors and recipients. Many tried, failed, and abandoned the field, but Thomas believed in the potential of this treatment. In the mid–late 1960s, methods to identify and type human leukocyte antigens (HLA) in humans were developed,^13^ which allowed for donor and recipient HLA matching. In 1969 Thomas initiated a clinical trial program in Seattle for allogeneic HSCT. In 1977, the Seattle group reported 100 transplantations, with chemotherapy and radiation therapy in 54 patients with acute myeloid leukemia (AML) and in 46 patients with acute lymphoblastic leukemia (ALL). Only 13 patients were alive without disease 1–4.5 years after HSCT.^14^ However, this small cure rate only encouraged Thomas to try and apply allogeneic HSCT earlier in the course of acute leukemia, and in 1979 he reported a cure rate of 50% in AML patients transplanted in first remission.^15^ Perhaps the most important thing Thomas found in his work was the power of the immune system to eradicate cancer. In 1990, E. Donnall Thomas won a Nobel Prize for his discoveries in cell transplantation in the treatment of human disease.

Another breakthrough took place with the first transplantation done from an HLA-matched unrelated donor (MUD).^16^ Hematopoietic stem cell transplantation from an unrelated donor dramatically increased the odds for finding a match; for example, it rose from 25% to 75% for Caucasian patients.^17^ International collaboration was mandatory for the establishment of transplantation centers around the world and for a global donor registry. In 1972 the International Bone Marrow Transplant Registry (IBMTR) was established for documenting HSCT outcome data. By that time, transplantations were done in 12 centers performing about 50 procedures a year altogether. In 1974, the European Group for Blood and Marrow Transplantation (EBMT) was established for European collaboration in the field of HSCT. The first unrelated donor transplantation inspired in 1986 the foundation of the National Marrow Donor Program (NMDP), and in 1988 Bone Marrow Donors Worldwide (BMDW) was founded. This organization unifies more than 23 million donors registered in 73 countries and 600,000 cord blood units from cord blood banks in 32 countries.^18^

## CURRENT STATUS OF HSCT

### Trends in Indications for HSCT

Autologous HSCT accounts for 58% of the transplantations done in Europe today;47% of the autologous HSCT are performed for multiple myeloma, 30% for non-Hodgkin lymphoma, 11% for Hodgkin lymphoma, and 3% for leukemia. Other less common indications for autologous HSCT include autoimmune disease (multiple sclerosis, systemic sclerosis, and Crohn’s disease) and solid tumors (sarcoma, germinal tumors, and neuroblastoma). Acute myeloid leukemia and ALL account for 50% of the allogeneic HSCT, myelodysplastic syndrome and myeloproliferative neoplasms account for 15%, and bone marrow failure syndrome for 6%. Other less common indications for allogeneic HSCT include lymphoma, myeloma, and hematologic disorders like aplastic anemia and thalassemia.^6^ Indications for HSCT have changed over time. Metastatic breast carcinoma was a major indication for autologous HSCT in the 1990s, but eventually well conducted randomized trials showed no benefit of the procedure, and today only a few cases a year are performed worldwide.^19^ In 2001, the tyrosine kinase inhibitor imatinib mesylate revolutionized the treatment of chronic myeloid leukemia (CML), and from a leading indication for allogeneic HSCT it now turned into a rare one, with allogeneic HSCT performed only in CML patients resistant to therapy or in transformation to acute leukemia.^20^ In 1982, allogeneic HSCT was first used for the treatment of thalassemia, and in 1984 for the management of sickle cell disease. Other novel indications have emerged like inherited metabolic disorders accounting for over 1,000 allogeneic HSCT between 1980 and 2006 and currently accounting for almost 1% of allogeneic HSCT in Europe.^21^ Autoimmune disease (AID) accounts for 1% of autologous transplantations and 0.1% of the allogeneic transplantations in Europe.^4^ Since 1996, as many as 1,300 autologous HSCT for AID have been registered in the EBMT. Autologous HSCT is most commonly performed in patients with non-responding multiple sclerosis or systemic sclerosis. Systemic lupus erythematosus, Crohn’s disease, type I diabetes, and juvenile idiopathic arthritis are other investigational indications.^22^ Change in indications or timing of HSCT may emerge in multiple myeloma, where upfront autologous transplantation has been the standard of care for many years. With the use of new drugs in myeloma (e.g. lenalidomide) the approach of early HSCT has been challenged by a new approach of delayed HSCT when disease progresses. To date, the results still show an advantage in progression-free survival and overall survival for patients undergoing early autologous HSCT.^23^ However, with the upcoming use of new and more potent drugs (e.g. carfilzomib, pomalidomide, daratumumab—an anti-CD38 antibody), the role of autologous HSCT in myeloma might be challenged again. Current indications could also change when better therapies emerge, and new indications appear, but time will be needed to confirm their benefits (Table 1).

**Table 1.** Current Indications for Autologous and Allogeneic Stem Cell Transplantation.

	Autologous Transplantation^*^	**Allogeneic Transplantation^†^**
Malignancies	Multiple myeloma	Acute myeloid leukemia
	Non-Hodgkin lymphoma	Acute lymphoblastic leukemia
	Hodgkin disease	Chronic myeloid leukemia
	Acute myeloid leukemia	Myelodysplastic syndromes
	Neuroblastoma	Myeloproliferative neoplasms
	Ovarian cancer	Non-Hodgkin lymphoma
	Germ-cell tumors	Hodgkin disease
		Multiple myeloma
		Juvenile chronic myeloid leukemia
Non-malignant disorders	Autoimmune disease	Aplastic anemia
	Amyloidosis	Paroxysmal nocturnal hemoglobinuria
		Fanconi’s anemia
		Diamond-Blackfan anemia
		Thalassemia major
		Sickle cell anemia
		Severe combined immunodeficiency
		Wiskott–Aldrich syndrome
		Inborn errors of metabolism
		Congenital neutropenia syndromes

* More than 30,000 autologous transplantations are performed annually worldwide, two-thirds for multiple myeloma or non-Hodgkin lymphoma.

† More than 24,000 allogeneic transplantations are performed annually worldwide, more than half for acute leukemias.

### Trends in HSCT Conditioning—from Chemotherapy to Immunotherapy

**Figure 1.**
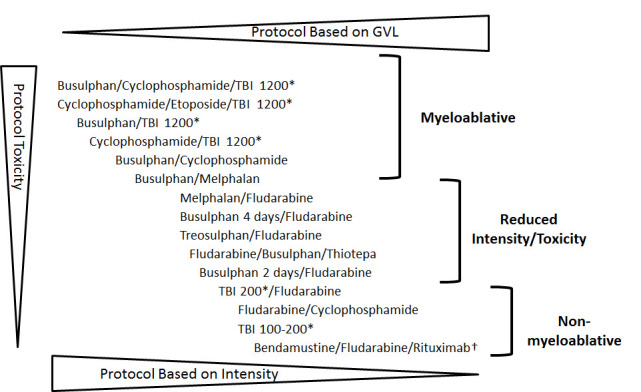
**Conditioning Regimen Intensity.** The more intense and myeloablative the protocol, the more toxic it is and less in need to rely on graft-versus-leukemia (GVL) for residual disease elimination. Reduced-intensity regimens are less toxic and rely more on an immunotherapeutic GVL effect to prevent relapse. Conditioning may include also the use of anti-thymocytic globulins in MUD HSCT. HSCT, hematopoietic stem cell transplantation; MUD, matched unrelated donor; TBI, total body irradiation. * The number represents the radiation dose in Rads. †New conditioning in phase II trial for chronic lymphocytic leukemia patients.^24^

The preparative regimen of chemotherapy or combined chemotherapy and radiotherapy prior to stem cell transfusion has a role in eradicating residual tumor and suppressing the patient’s immune system to prevent graft rejection. There are specific regimens for each indication based on data from clinical trials. The regimens are categorized by their intensity level as full myeloablative, reduced-toxicity or reduced-intensity and non-myeloablative ones (Figure 1). The initial allogeneic HSCTs were based on total body irradiation (TBI) at doses of 1,000–1,600 Rad. Cyclophosphamide (Cy) was added later to increase both anti-tumor activity and immune suppression, which facilitated engraftment.^14^ It was further found that cyclophosphamide and busulfan provided a lower transplant-related mortality (TRM) and a better disease-free survival in AML compared to the TBI/Cy regimen.^25^ The high rate of toxicity and TRM limited the use of these regimens to younger and fit patients up to the age of 50 years.

The breakthrough occurred due to the understanding that there is an immunologic response of the hematopoietic graft against hematologic malignancy. The idea of graft-versus-leukemia (GVL) was supported by observations that transplantation from a syngeneic twin and the use of T-cell-depleted grafts were associated with a higher relapse rate, while occurrence of graft-versus-host disease (GVHD) was associated with a decreased relapse rate.^26^ Under the assumption that augmentation of the graft immune activity can restore remission after relapse, in 1988, the first three patients were treated with donor leukocyte infusion (DLI) for relapsed CML after allogeneic HSCT and indeed achieved a second remission.^27^ This form of cellular immunotherapy has evolved, and today DLI is a routine procedure used after allogeneic HSCT for treating incomplete engraftment, relapsed disease, and as pre-emptive therapy to prevent relapse.^28^ Following these new insights on the immunotherapy aspects of the allogeneic HSCT, it was first reported in 1998 that reduced-intensity conditioning (RIC) could provide sustained engraftment and eradicate hematologic malignancy and patient’s hematopoiesis.^29^ The conditioning was based on anti-lymphocyte drugs, to suppress the recipient T cells and thus prevent graft rejection, in combination with a reduced dose of anti-tumor chemotherapy in order to reduce the toxicity and TRM. The use of RIC and later application of non-myeloablative conditioning allowed for extending the application of allogeneic HSCT for older and medically unfit patients.^30^,^31^ Furthermore, the RIC HSCT combined with progress in the supportive care allows today for safer transplantation even in patients up to the age of 70 years.

### Extending Donor Availability

Apart from possibilities of a matched related sibling donor and a matched unrelated volunteer donor, it has become optional to perform allogeneic HSCT for almost all patients using an alternative donor. The alternatives are mismatched unrelated donor (MMURD), haploidentical related donor, and umbilical cord blood (UCB) transplantation. A usual accepted alternative is MMURD if matched at least in 7/8 HLA alleles (HLA A, B, C, and DRB1) in high-resolution typing techniques.^32^ Otherwise, if there is more than one mismatch the TRM risk is significantly increased compared to allogeneic HSCT performed from a fully matched unrelated donor.

In haploidentical transplantation, the donor is a first-degree relative (parent, sibling, or child) partially matched to the patient, at least in one haplotype. Haploidentical transplantation initially used full marrow grafts and was associated with unacceptably high rates of lethal GVHD. Later on, T cell depletion (TCD) of grafts was used to reduce this risk, but rates of engraftment failure were high. Eventually, in 2005 a pilot phase II study reported haploidentical transplantation using graft TCD combined with a “megadose” of stem cells (a median of 13.8 million stem cells per kg of patient weight compared to a median of 5 million needed for matched related donor (MRD) or MUD transplantation) which proved able to improve engraftment rates.^33^ However, the main limitation of haploidentical transplantation still remains a higher rate of non-engraftment compared to MRD or MUD, and a significant rate of TRM due to infections caused by delayed immune reconstitution.^34^

Umbilical cord blood transplantation is an established alternative source of stem cells in the last 25 years. The naive properties of cord immune cells allows for the UCB unit to be matched with the recipient in only 4–6/6 HLA alleles (HLA A and B in antigenic level and HLA DRB1 in allelic level). However, the use of UCB is limited due to the low number of stem cells in a given cord unit relative to an adult’s weight.^35^ Different approaches have been applied to increase the number of stem cells in the UCB, including the use of double cord blood units and *ex vivo* expansion of the cord unit. The major advantage of UCB transplant is its relative accessibility as it is a “shelf” product. The disadvantages of this transplant are related to the higher engraftment failure rate and an increased risk of immunosuppression and infection complications due to the naivety of the cells with no prior exposure to different pathogens. Additionally, there is no donor available for a later use of DLI. Eventually, in a large-scale analysis of retrospective and observational data, the disease-free survival and overall survival were found to be similar in transplants using different alternative donors, but inferior compared to matched donor (MRD or MUD) transplants.^36^

The availability of donors also increased due to the shift from collecting stem cells by bone marrow aspiration in the operation room to peripheral blood stem cell collection. Excessive stem cell production is stimulated by injecting recombinant granulocyte colony-stimulating factor (G-CSF) to the donor. Stem cells are then collected by leukopheresis using an apheresis machine, which is an automated, continuous flow blood cell and plasma components separator. This process was first described in 1993 in a very small number of patients and their donors.^37^ Stem cells collected from peripheral blood proved to be safe for the donors and efficient for the patients (regarding overall survival) when compared to bone marrow-harvested stem cells. Nowadays, almost 80% of allogeneic transplantations in adult patients over the age of 20 years are performed using peripheral blood stem cells.^6^

### Outcomes—Reducing Transplant-Related Morbidity and Mortality

The major limitation of allogeneic HSCT remains the high rate of mortality and morbidity. The mortality rate at 100 days post allogeneic HSCT ranges between 7% for patients with acute leukemia in remission undergoing MRD HSCT and 27% for patients with refractory acute leukemia undergoing MUD HSCT. The main TRM reasons are GVHD (17% in MRD HSCT, 19% in MUD HSCT) and infections (12% in MRD HSCT, 17% in MUD HSCT).^6^ Patients who survive for 2 years without disease relapse have a probability for long-term survival of 80%–92%. However, their life expectancy continues to lag behind that of their age- and gender-matched peers from the general population for 15–20 years after the transplantation. Even in disease-free patients in the first 2 years after transplantation, disease relapse still accounts for 41% of late mortality, followed by chronic GVHD, infections, organ failure, and secondary cancers.^38^

The currently observed decrease in TRM is mainly attributed to the improvement in supportive care. The use of RIC and non-myeloablative regimens has been proved effective in high-risk disease like acute leukemia, with fewer toxicities and complications during the transplantation and a similar long-term overall survival compared to myeloablative conditioning.^39^ The development of specific clinical scoring systems to predict better the patient TRM risk allows for a personalized risk-adapted decision-making regarding HSCT.^40^,^41^ Strategies for pre-emptive detection and therapy of severe infectious complications such as cytomegalovirus disease and invasive aspergillosis have become standard of care.^42^–^44^ However, both disease relapse and GVHD remain major causes of mortality with limited improvement over recent years. In spite of prophylaxis and treatment, high-grade acute GVHD still occurs in about 11%–18% of patients with a mortality rate of 70%–90% in its severe form.^45^,^46^ Hematologic malignancy relapse after allogeneic HSCT has very limited treatment options and a poor prognosis. In relapsed acute leukemia the 2-year survival rate is 14%–16%, and the most effective treatment strategy is to achieve second remission with chemotherapy followed by consolidation with DLI or second allogeneic HSCT.^47^,^48^

## FUTURE PERSPECTIVES

In recent years, research has focused on strategies for decreasing disease relapse rate. Methods manipulating the graft immune activity towards GVL with minimizing GVHD represent a new exciting direction which could promote the next breakthrough in allogeneic HSCT. Graft-versus-leukemia is mainly attributed to the immune response of donor T lymphocytes against residual or relapsed leukemic cells, as well as to the immune response of natural killer (NK) cells and B lymphocytes.^49^ Among the leading strategies that have already reached the clinical phase of investigation are graft manipulations in the setting of haploidentical HSCT, vaccinations against tumor-associated antigens (TAA), monoclonal antibodies, and targeted adoptive cellular immunotherapy.

### Regulatory T Cell Infusion

Haploidentical HSCT allows for immediate donor availability for 95% of patients. However, its use has been limited by a significant rate of non-engraftment and infections.^32^ One strategy to overcome these issues is to engineer the graft and replete it with regulatory and effector T cells in a specific ratio. Human natural regulatory T cells (Tregs) derive from the thymus and express high levels of CD25 (interleukin 2 receptor alpha) and intracellular Foxp3 (forkhead box P3) which is a master switch transcription factor.^50^,^51^ Tregs suppress other active conventional T cell (Tcon) populations allowing for immune homeostasis and therefore have a role in preventing autoimmunity and limiting inflammatory disease.^52^ Tregs were shown to be capable of inducing immune tolerance in animal models of bone marrow transplantation, thus preventing GVHD without hampering GVL.^53^–^55^ To date, several phase I–II clinical trials have been conducted. One study was performed in the setting of double UCB transplantation, in which third-party UCB Tregs were *ex vivo* expanded and infused. The procedure was shown to be safe with no increased rates of GVHD, infections, or disease relapse.^56^ In another study pre-emptive Treg DLI was performed after stopping immunosuppression, in order to prevent relapse after allogeneic HSCT in high-risk patients.^57^ In one study, Treg DLI from a donor was given as treatment for active chronic GVHD after allogeneic HSCT.^58^ In two other studies in the haploidentical HSCT setting, Tregs were infused a few days prior to the transfusion of a TCD stem cell graft combined with Tcon infusion. The Treg and Tcon ratio was 2:1, and the purpose of the study was to use the Tregs as the sole GVHD prophylaxis strategy and as a way to improve immune reconstitution without increasing disease relapse rate.^59^,^60^ While GVHD and relapse rates in these studies were lower compared to historical controls, non-relapse mortality is still unsatisfactory.

### Graft Depletion of αβ CD19 T Cells

The αβ T cell receptor (TCR)-positive T cells constitute 95% of the T cell population. These lymphocyte subsets are responsible for the occurrence of GVHD.^61^ γδ T cells (also termed “innate-like” T cells or “transitional” T cells) belong to the adoptive arm of the immune system.^62^ They are capable of recognizing their targets in a major histocompatibility complex-independent manner; therefore, they are unlikely to elicit GVHD. Higher levels of γδ TCR-positive T cells were found to correlate with better leukemia-free survival in haploidentical HSCT recipients.^63^ This finding led to two phase I–II clinical trials in the setting of haploidentical HSCT. Grafts were depleted from αβ T cells, allowing for the transplantation of γδ lymphocyte retained grafts. No other prophylactic treatment was given for GVHD prevention.^64^,^65^ This method allowed a sustained engraftment, rapid immune reconstitution, and low incidence of both acute and chronic GVHD with a comparable non-relapse mortality rate to that of MUD. Regarding disease relapse the follow-up is still too short.

### Natural Killer Cell Adoptive Immunotherapy

In TCD haploidentical HSCT it was shown that mature fully functioning NK cells derived from differentiation of hematopoietic stem cells emerge in the peripheral blood of the recipient only several weeks after the allograft, while in the early post-transplant period immature poorly functioning NK cells predominate.^66^ Therefore patients receiving allografts from haploidentical NK alloreactive relative donors cannot benefit from NK-mediated GVL effect in the early post-transplant period. This led to a phase II trial of pre-emptive infusion of donor NK cells (day 4, day 30, and day 100 post-transplant) with an aim of preventing graft failure and adding to GVL effect without increasing GVHD.^66^ Compared to historical controls there was no advantage in preventing graft failure or disease relapse. However, this treatment is safe and feasible and should be further explored regarding NK cell dose, timing, and need for activation of the NK cells prior to their transfusion.

### Anti-Tumor Vaccination

Autologous primed T cells targeted against specific tumor-associated antigens or against the whole tumor cell have been developed and tested in acute myeloid leukemia (Wilms tumor-associated antigen 1, WT1), chronic myeloid leukemia (BCR-ABL peptide epitopes), and multiple myeloma (dendritic cell/myeloma fusion cells). Many trials have tested augmenting the patient immune response against the tumor in the autologous setting.^68^,^69^ In two trials, autologous inactivated leukemia cells were transfused to patients following allogeneic stem cell transplantation to induce *in vivo* immunity. The procedure was proved to be safe, immunogenic, and associated with biological activity despite the use of immunosuppression for GVHD prophylaxis.^70^,^71^ However, this treatment may have significant advantage in the setting of allogeneic HSCT allowing for the transfer of targeted adoptive immunotherapy specific against the tumor with no risk for GVHD. By this approach vaccinating the donor before lymphocyte collection has the advantage of vaccine-primed lymphocytes collected from a healthy donor with healthy immune system, rather than from patients tolerant to their own tumor antigens with reduced immunity from prior chemotherapy. It has been tested in a small number of patients with multiple myeloma, where the donors were immunized with the recipient myeloma paraprotein. In one case the donor was immunized prior to DLI collection to treat relapsed disease after the HSCT.^72^ In the other cases the donor was immunized before stem cell collection, and the recipient showed immunogenic response with long-term remission.^73^,^74^ Further trials are required to find immunogenic targets and immunization strategies in the allogeneic HSCT setting. The main limitation is due to the need to perform the vaccination procedures on a healthy donor. Priming and expanding donor T cells *ex vivo* against patient TAA will resolve the issue of vaccinating the patient himself; however, this will require very high costs.

### Monoclonal Antibodies

Monoclonal antibodies (mAb) are in routine use in the treatment of hematologic malignancies. Rituximab, an anti-CD20 antibody, is the standard treatment protocol for B cell lymphomas. Gemtuzumab ozogamicin, a humanized anti-CD33 mAb conjugated to calicheamicin-derivate toxin, has been used in AML and been shown when incorporated in low dose with chemotherapy to result in improved overall survival.^75^ Brentuximab vedotin, an anti-CD30 mAb linked to monomethyl auristatin E, a microtubule-disrupting agent, has revolutionized treatment in Hodgkin lymphoma and CD30-positive peripheral T cell lymphoma.^76^ Now in preclinical and clinical trials are the bispecific T cell engager antibodies (BiTEs). These antibodies bind target tumor cells and at the same time bind and harness polyclonal cytotoxic T cells to cause highly efficient lysis of targeted tumor cells. Blinatumumab, an anti-CD3/CD19 BiTE mAb, has been tested in relapsed and refractory B cell acute lymphoblastic leukemia patients.^77^ Other CD3/CD33 BiTEs have shown excellent results in preclinical trials for AML.^78^,^79^ New alternative antigenic targets in AML are being investigated.^80^ The role of these mAb in the setting of allogeneic HSCT has yet to be determined.

### Chimeric Antigen Receptors

**Figure 2.**
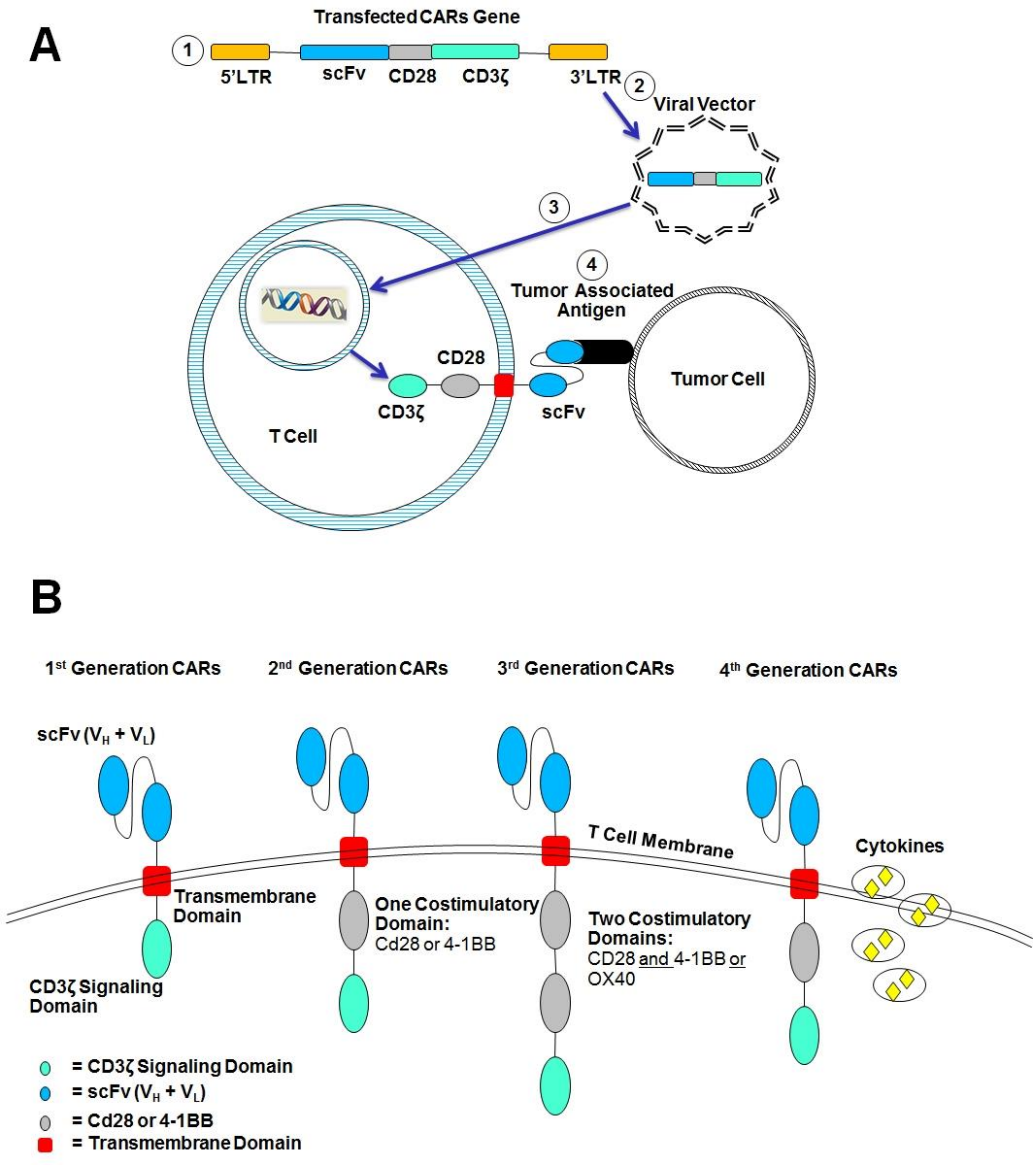
**The Chimeric Antigen Receptor (CAR).** **A: Construction and function of the CAR.** ⓵ Constructed gene contains the tumor-associated antigen binding site (scFv), a co-stimulatory region (e.g. CD28), and an activating signal region (CD3ζ). ⓶ The gene is transfected into the T cell using a viral vector. ⓷ CAR gene incorporates into the cell DNA and translates into CAR protein. ⓸ CAR binds to the tumor-associated antigen (TAA), and the T cell is activated to cause tumor cell lysis, to secrete cytokines, and to proliferate. **B: CARs transfected gene encodes to an extra-membrane TAA binding domain (scFv), a transmembrane domain and endomembrane cell activating domain (CD3ζ)**. First-generation CARs contain one signaling domain, the cytoplasmic signaling domain of the CD3 TCRζ chain. Second-generation CARs contain the activating domain and a co-stimulatory domain, typically the cytoplasmic signaling domains of the co-stimulatory receptors CD28 and 4-1BB or OX40. Third-generation CARs harness the signaling potential of two co-stimulatory domains: CD28 domain followed by either the 4-1BB or OX40. Fourth-generation CARs may be further enhanced through the introduction of additional genes, including those encoding proproliferative cytokines (e.g. IL-12).

Chimeric antigen receptors (CARs) are recombinant receptors that provide both antigen-binding and T cell-activating functions (Figure 2). The engineering of CARs into T cells requires that T cells be cultured to allow for gene transduction and stable clonal expansion. Any cell surface molecule can be targeted through a CAR. Current CARs are limited to recognizing only cell surface antigens (T cell receptors recognize both cell surface and intra-cellular proteins). However, CARs do not require antigen processing and presentation by HLA. Therefore CARs recognize antigen on any HLA background, in contrast to T cell receptors (TCR), which need to be matched to the patient’s haplotype. Furthermore, CARs can target tumor cells that down-regulate HLA expression or use proteasomal antigen processing, two mechanisms that contribute to tumor escape from TCR-mediated immunity.^81^ The most investigated target to date is CD19 found on B cell lymphocyte and on malignancies arising from it (B cell non-Hodgkin lymphoma, chronic lymphocytic leukemia, B cell acute lymphoblastic leukemia). Several phase I–II studies have been conducted in patients with very refractory disease, with some of the studies showing promising results.^82^–^85^ Novel targets are being investigated in preclinical setting like the promising CARs against CD123, which is found on acute myeloid leukemia cells.^86^,^87^ Chimeric antigen receptor T cell adoptive therapy seems to have a great potential, and its best effect might be in the allogeneic HSCT setting.

### Selective Allo-Depleted T Cells

**Figure 3.**
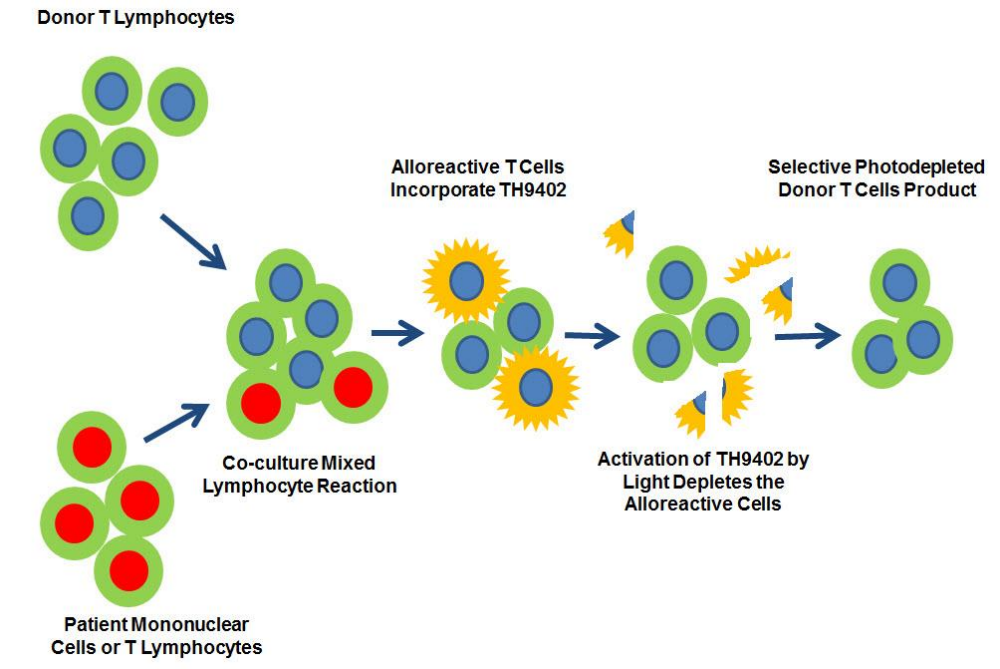
**Alloreactive T Cell Photodepletion.** Stimulator mononuclear/T cells are collected from the patient by leukopheresis. They are expanded in culture (with IL-2 and OKT3), and before use they are inactivated by irradiation. The cells are co-cultured in a 1:1 ratio with donor T cells collected by leukopheresis. The cells are then incubated with the photosensitizer TH9402. Alloactivated T cells incorporate the photosensitizer. Activation of TH9402 by light exposure causes their lysis and allodepletion of the alloreactive lymphocytes. Only non-alloreactive donor T cells remain in the product.

T cell depletion abrogates GVHD completely with higher rates of non-engraftment, infections, and disease relapse. Host alloreactive donor lymphocytes are responsible for GVHD. Theoretically selective elimination of host alloreactive lymphocytes from the graft will result in significant reduction in GVHD rate without effecting GVL and immune reconstitution. Selective allodepletion methods rely on the co-culturing of irradiated host peripheral blood mononuclear cells with donor T cells. Alloreactive T cells are activated, identified, and removed either with monoclonal antibodies coupled to magnetic beads or photodepletion procedure (Figure 3). Peripheral blood stem cells are collected from the donor with leukopheresis. The graft is then T cell-depleted by positive stem cell selection. The unabsorbed T cells are used for the selective depletion. On the transplantation day the patient receives the graft and the selective allodepletion T cells.^88^ Few clinical trials have been carried out to prove the concept.^89^,^90^ However, the procedure is laborious and expensive, and necessitates a “good manufacturing practice” facility.

### Antiviral Cytotoxic Cell Lines

Viral infections are a significant cause of morbidity and mortality in transplantation, especially in pediatric patients, and particularly in TCD haploidentical transplantation and in UCB transplantation. Effective therapies are limited in refractory infections and often associated with significant side effects. Adoptive transfer of virus-reactive T cells offers a means of reconstituting antiviral immunity, and this approach has been successfully used to prevent and treat cytomegalovirus, Epstein–Barr virus, and adenovirus infections. Adoptive antiviral cytotoxic T cell lines have been used effectively for more than a decade. However, this therapy necessitates a “good manufacturing practice” facility.^91^

## CONCLUSIONS

In December 2012, the one-millionth blood stem cell transplant worldwide was performed. In the last decade, 30%–40% of all transplantations were allogeneic and outcomes have significantly improved. Reduced-intensity conditioning better exploiting the immunotherapeutic GVL effect and improved supportive care have contributed to reduction in TRM rate. Recipient age is rising, and now HSCT is considered optional up to the age of 70 years. Donor availability has dramatically increased thanks to the international collaboration and unrelated volunteer donor registries. Haploidentical HSCT holds the potential to find a donor for almost all patients, and new strategies for improving the transplant outcome, like Tregs infusion and αβ T cell depletion, are being investigated in clinical trials. The use of adoptive cellular immunotherapy, tumor vaccinations, and mAbs is expected to change the allogeneic HSCT setting and reduce disease relapse rate. In the coming years allogeneic HSCT is likely to become more complex, more individualized, and more efficient.

## Abbreviations:

AID: autoimmune disease;
ALL: acute lymphoblastic leukemia;
AML: acute myeloid leukemia;
BiTEs: bispecific T cell engager antibodies;
BMDW: Bone Marrow Donors Worldwide;
CML: chronic myeloid leukemia;
CARs: chimeric antigen receptors;
Cy: cyclophosphamide;
DLI: donor lymphocyte infusion;
EBMT: European Group for Blood and Marrow Transplantation;
G-CSF: granulocyte colony-stimulating factor;
GVHD: graft-versus-host disease;
GVL: graft-versus-leukemia;
HLA: human leukocyte antigen;
HSCT: hematopoietic stem cell transplantation;
IBMTR: International Bone Marrow Transplant Registry;
mAb: monoclonal antibodies;
MMURD: mismatched unrelated donor;
MRD: matched related donor;
MUD: matched unrelated donor;
NMDP: National Marrow Donor Program;
RIC: reduced-intensity conditioning;
TAA: tumor-associated antigens;
TBI: total body irradiation;
Tcon: conventional T cell;
TCD: T cell depletion;
TCR: T cell receptor;
Treg: regulatory T cell;
TRM: transplant-related mortality;
UCB: umbilical cord blood.
